# Foodborne Cereulide Causes Beta-Cell Dysfunction and Apoptosis

**DOI:** 10.1371/journal.pone.0104866

**Published:** 2014-08-13

**Authors:** Roman Vangoitsenhoven, Dieter Rondas, Inne Crèvecoeur, Wannes D'Hertog, Pieter Baatsen, Matilde Masini, Mirjana Andjelkovic, Joris Van Loco, Christophe Matthys, Chantal Mathieu, Lut Overbergh, Bart Van der Schueren

**Affiliations:** 1 Laboratory for Clinical and Experimental Medicine and Endocrinology, KU Leuven, Leuven, Belgium; 2 EM Facility, VIB Bio Imaging Core and VIB department for the Biology of Disease, KU Leuven, Leuven, Belgium; 3 Department of Translational Research and of New Surgical and Medical Technologies, University of Pisa, Pisa, Italy; 4 Food, Medicines, and Consumer Safety, Scientific Institute of Public Health, Brussels, Belgium; University of Alabama at Birmingham, United States of America

## Abstract

**Aims/Hypothesis:**

To study the effects of cereulide, a food toxin often found at low concentrations in take-away meals, on beta-cell survival and function.

**Methods:**

Cell death was quantified by Hoechst/Propidium Iodide in mouse (MIN6) and rat (INS-1E) beta-cell lines, whole mouse islets and control cell lines (HepG2 and COS-1). Beta-cell function was studied by glucose-stimulated insulin secretion (GSIS). Mechanisms of toxicity were evaluated in MIN6 cells by mRNA profiling, electron microscopy and mitochondrial function tests.

**Results:**

24 h exposure to 5 ng/ml cereulide rendered almost all MIN6, INS-1E and pancreatic islets apoptotic, whereas cell death did not increase in the control cell lines. In MIN6 cells and murine islets, GSIS capacity was lost following 24 h exposure to 0.5 ng/ml cereulide (P<0.05). Cereulide exposure induced markers of mitochondrial stress including *Puma* (p53 up-regulated modulator of apoptosis, P<0.05) and general pro-apoptotic signals as *Chop* (CCAAT/-enhancer-binding protein homologous protein). Mitochondria appeared swollen upon transmission electron microscopy, basal respiration rate was reduced by 52% (P<0.05) and reactive oxygen species increased by more than twofold (P<0.05) following 24 h exposure to 0.25 and 0.50 ng/ml cereulide, respectively.

**Conclusions/Interpretation:**

Cereulide causes apoptotic beta-cell death at low concentrations and impairs beta-cell function at even lower concentrations, with mitochondrial dysfunction underlying these defects. Thus, exposure to cereulide even at concentrations too low to cause systemic effects appears deleterious to the beta-cell.

## Introduction

The prevalence of type 1 and type 2 diabetes is rising and this increase is believed to be related to environmental factors. Next to sedentary lifestyle and western style diet, it is known that certain environmental polluents, such as polychlorinated biphenyls, xenoestrogens or cadmium can cause beta-cell dysfunction and ultimately cell death, both key traits of the pathophysiology of diabetes [1], [2] .

Thus, foodborne toxins, ubiquitous in this era of prepackaged and take-away meals, might contribute to the current rise in diabetes prevalence. Here, we propose cereulide, a toxin produced by certain strains of *Bacillus cereus*, as a putative culprit. It is a cyclic dodecadepsipeptide (1.2 kDa), that acts as a potassium ionophore, and is structurally very similar to the well-known valinomycin. Cereulide has been shown to uncouple oxidative phosphorylation by permeabilizing the mitochondrial membrane [3]. As a result, cereulide inhibits cell proliferation, RNA synthesis and motility in different cell types [4]–[6]. As mitochondrial ATP production is a key element for glucose-stimulated insulin production, cereulide's mitochondriotoxic properties might be specifically harmful for beta-cell function and survival. Exposure of porcine pancreatic islets to 1 ng/ml of cereulide, decreased insulin content and increased necrotic cell death within 2 days [7].

Cereulide is highly resistant to heat, acidity and proteolysis, which renders it difficult to eradicate from the food chain and prevents its neutralization in the digestive tract. Moreover, the highly lipophilic character of cereulide (log Kow 5.96) suggests possible bioaccumulation in human tissues which could further increase the potential health hazard [8], [9].

Cereulide is well known to cause acute and possibly fatal emetic food poisoning, with an estimated lethal dose of 8 µg/kg body weight [10]. Most of the cases of acute toxicity involved the liver (fulminant hepatic failure) and cereulide has been detected in the gut, peritoneum and spleen of intoxicated patients [11]. Shiota et al. have been able to detect 4 ng/mL cereulide in plasma of 2 children presenting with emetic food poisoning [12], which supports the hypothesis that cereulide enters both the portal and systemic circulation.

Chronic exposure to low concentrations of cereulide might have a more devious effect. The exact prevalence of cereulide toxin in food remains uncertain, mainly because a full diagnostic workup is seldom performed in self-limiting gastro-intestinal syndromes, and a simple and cheap diagnostic test is not available. Almost all of the available data are derived from dramatic - and sometimes fatal – cases of acute food poisoning. The prevalence of the *Bacillus cereus* infections (all strains combined) is rising throughout Europe [13]. Agata et al. report concentrations of 0.01 to 1.28 µg/g in 13 out of 14 food samples, and others have measured concentrations up to 13.2 ng/g food [14], [15]. Very recently, Messelhäusser and colleagues, investigated 4300 food samples linked to foodborne illness over a 7 year period in Bavaria, and confirmed that cereulide is not only present in cooked rice dishes and pasta, but also in infant meals, cauliflower and milk [16]. Our group has shown that 7.4% of rice dishes randomly collected from Asian restaurants in Belgium, contained low concentrations of cereulide (4 ng/g food) upon sampling and this prevalence increases to 12.9% when food is exposed to temperature abuse conditions (25°C) [17]. Thus, although large scale prevalence data for the cereulide toxin itself are lacking, repeated exposures to nanogram levels of cereulide through food are likely. Up until now, little is known about the effects of exposure of beta-cells to low concentrations of the toxin. In this study, we set out to investigate whether beta-cells are in particular sensitive to cereulide and if so, what the underlying mechanisms are.

## Materials and Methods

### Cereulide toxin

Natural cereulide was extracted from *Bacillus cereus* (internal strain name Bieva) on tryptone soya agar (Oxoid CM 131, Erembodegem, Belgium), incubated at 30°C for 48 h and suspended in ethanol. The concentration was determined by LC-MS, as described before [18]. Unless stated otherwise, all experiments were conducted after 24 h exposure to cereulide concentrations of 0.15 ng/ml, 0.25 ng/ml or 0.5 ng/ml. Control conditions consisted of corresponding concentrations of ethanol, and gave no measurable effect.

### Cell culture

MIN6 cells, a kind gift from Dr. Jun-ichi Miyazaki (Institute for Medical Genetics, Kumamoto University Medical School, Kumamoto 862, Japan, [19]), were maintained at 5% CO_2_ and 37°C in DMEM with 25 mM glucose, supplemented with 15% heat-inactivated fetal bovine serum, 50 U/ml penicillin, 50 µg/ml streptomycin, 4 mM GlutaMAX, and 70 µM β-mercaptoethanol (all from Invitrogen-Gibco, Ghent, Belgium). MIN6 cells used for experiments ranged from passages 22 to 36. INS-1E cells (a kind gift from Prof. C. Wollheim, Centre Medical Universitaire, Geneva, Switzerland, [20]), HepG2 (ATCC HB-8065, Rockville, MD) and COS-1 (ATCC CRL­1650, Manassas, VA) cell lines were cultured as described before [21], [22].

### Pancreatic islet isolation

Whole mouse pancreatic islets were isolated from 2–3 weeks old C57Bl/6J mice (Charles River Laboratories, Brussels, Belgium) as described before [23]. Islets were handpicked under a stereomicroscope, and cultured in RPMI 1640 supplemented with 10% heat-inactivated fetal bovine serum, 100 U/ml penicillin, 100 µg/ml streptomycin, 2 mM GlutaMAX (all from Invitrogen-Gibco). Principles of laboratory animal care (NIH publication no. 85–23, revised 1985) were followed and all animal experimental procedures were approved by the Ethics Committee of the University of Leuven (permit number P056/2013). Animals were sacrificed by carbon dioxide gas, and all efforts were made to minimize suffering.

### Cell death assay

The percentage of living, apoptotic, and necrotic cells was assessed by microscopic cell counting. MIN6, INS-1E, HepG2 and COS-1 cells were cultured in 96-well plates. After 24 and 72 h of exposure to cereulide concentrations ranging from 0.05 ng/ml to 5 ng/ml, cells were incubated for 15 min with propidium iodide (PI, 20 µg/mL, Invitrogen) and Hoechst HO342 (4 µg/mL, Invitrogen) at 37°C. Whole mouse islet were exposed to cereulide concentrations ranging from 0.05 ng/ml to 5 ng/ml, for 24 hours. At least 500 cells, or 20 cultured islets, were evaluated in each experimental condition by two researchers, one of them unaware of the sample identity, on an inverted fluorescent microscope (Nikon, Brussels, Belgium).

### Caspase 3/7 activity

MIN6 cells were plated in a 96-well plate and exposed for 24 h to cereulide. Luminescence was measured on a Victor Wallac workstation (Perkin Elmer, Wallac, Finland), using the Caspase Glo 3/7 assay (Promega, Madison, WI), according to the manufacturer's instructions.

### Glucose-stimulated insulin secretion assay

Glucose-stimulated insulin secretion assay was performed in MIN6 cells and whole mouse islets after exposure for 24 h to different concentrations of cereulide (0.15; 0.25 and 0.5 ng/ml in MIN6 and 0.5 ng/ml in islets) or vehicle (ethanol). Cells or islets were washed and equilibrated in glucose-free Krebs-Ringer bicarbonate HEPES Buffer ((KRHB) containing 125 mM NaCl, 4.74 mM KCl, 1.2 mM KH_2_PO_4_·H_2_O, 1.2 mM, MgSO_4_·7H_2_O, 1 mM CaCl_2_·2H_2_O, 5 mM NaHCO_3_, 25 mM HEPES, and 0.1% BSA). The solution was then replaced with low glucose KRHB (2 mM for MIN6 and 3 mM for islets) or high glucose KRHB (20 M for MIN6 and 30 mM for islets), cells/islets were incubated for 1 h at 37°C. Supernatant as well as remaining insulin content (extracted with acidic ethanol) was recuperated for insulin determination using an anti-mouse insulin ELISA kit (Mercodia, Uppsala, Sweden) according to the manufacturer's instructions. Secreted insulin concentrations are expressed as percentage of total insulin content.

### Quantitative polymerase chain reaction

Total RNA was extracted using the High Pure RNA Isolation Kit (Roche, Basel, Switzerland) according to the manufacturer's instructions. After priming with oligo dT, 0.5 µg RNA was converted to single stranded complementary DNA using Superscript II RT (Life Technologies, Ghent, Belgium) at 42°C for 80 min. qRT-PCR was performed using a StepOne real-time PCR system (Applied Biosystems, Ghent, Belgium) and Fast SYBR Green Mastermix (Qiagen, Hilden, Germany), based on the ΔΔCt quantification method. Values were normalized to the geometric mean of housekeeping genes 60S ribosomal protein L27 and hypoxanthine-guanine phosphoribosyltransferase, whose expression was not influenced by experimental conditions. Melting curve analysis confirmed primer specificities. Primer sequences are listed in **table S1**.

### Electron microscopy

MIN6 cells were exposed to 0.5 ng/ml cereulide or vehicle (ethanol) only. After 24 h, cells were fixed with 2.5% glutaraldehyde in 0.1 M cacodylate buffer, scraped and pelleted in 1.5% agarose/cacodylate buffer, and post-fixed in cacodylate-buffered osmium tetroxide. After triple washing in 0.1 M cacodylate buffer, dehydration in a graded series of ethanol and *en bloc* staining with 3% uranyl acetate, samples were embedded in Agar100 epoxy resin and cured for 2 days at 60°C. Sections of 60 nm were cut with Leica UCT ultramicrotome and micrographs were taken from all cells in a section using a JEOL 1400 transmission electron microscope, equipped with a Olympus SIS Quemesa 11 Mpx camera at pixel sizes of 5.8 nm (2500x), 29 nm (500x). Morphometric analysis was performed by a stereological method [24]. Volume density was calculated according to the formula: Volume Density  =  Pi/Pt, where Pi is the number of points within the subcellular component and Pt is the total number of points, and expressed in ml/100 ml of tissue. 50 individual cells were evaluated by an experienced researcher, unaware of sample identity.

### Oxidative phosphorylation capacity

Oxygen consumption rate (OCR) was measured with the XF24 Analyzer and XF24 extracellular flux assay kit (Seahorse Bioscience Inc., MA). After allowing the cells to attach, they were exposed to 0.15 or 0.25 ng/ml cereulide for 24 h, the medium was replaced with assay medium (DMEM D5030, supplemented with 2 mM glutamine and 500 mM glucose, Sigma Aldrich, Diegem, Belgium) and equilibrated in a non-CO_2_ incubator for 1 h. Basal OCR was measured 5 times for 3 min every 10 min, followed by sequential injection of 6.3 mM Oligomycin, and 4 mM Antimycin A (both from Sigma Aldrich). OCR results were normalized to DNA content, to correct for differences in cell number per experimental condition.

### Reactive oxygen species (ROS)

Levels of cellular oxidative stress were measured using the fluorescent probe Dichlorofluorescindiacetate (DCFDA, Invitrogen). MIN6 cells were loaded during 30 min with DCFDA (10 µM in culture medium) at 37°C. Subsequently, medium was removed and cells were exposed to cereulide for 24 h. The formation of the fluorescent product DCF was determined on a Victor Wallac workstation and normalized to DNA content.

### Cytochrome c release – Western blotting

MIN6 cells were exposed to cereulide, washed in cold PBS, harvested by cell scraping in 1 ml PBS and centrifuged at 700×g for 2 minutes. Supernatant was removed, and the pellet was resuspended in 50 µL of permeabilizing buffer (75 mM NaCl, 1 mM NaH_2_PO_4_, 8 mM Na_2_PO_4_, 250 mM Sucrose, 21 µl/µl aprotinin, 1 mM PMSF and 0.8 µg/µl digitonin) containing protease inhibitors (Roche). Subsequently, samples were vortexed for 30 sec, and centrifuged for 1 min at 20 000×g. The supernatant is considered cytosolic fraction, which was mixed with an equal volume of 2 x loading buffer. Samples were loaded on a 4–12% Bis-Tris gel (Nu PAGE, Life technologies, Ghent, Belgium), electrophoresed, and transferred to a PVDF membrane (Hybond-ECL; GE Healthcare, Diegem, Belgium). Membranes were blocked with 5% bovine serum albumin (BSA) for 1 h, and incubated overnight with the primary antibody for anti-cytochrome C (1/1000, BD Biosciences, Erembodegem, Belgium) or anti-(phosphorylated) protein kinase RNA-like endoplasmic reticulum kinase PERK (1/1000, Cell signaling, Erembodegem, Belgium). Membranes were incubated for 1 h with horseradish peroxidase-conjugated species-specific secondary antibody (1/3000, DAKO, Glostrup, Denmark), washed in TBS-Tween and were developed using Western lightning Plus-ECL detection system (PerkinElmer, Zaventem, Belgium) and read on an Image Quant LAS 500 (GE Healthcare) .

### Statistical analysis

Data are presented as mean ± SEM. Statistical analyses were performed with GraphPadPrism 5.0 for Windows (GraphPad Software, San Diego, CA). Data were compared by one way Analysis of variance (ANOVA) followed by post hoc analysis with Dunnet's multiple-comparison test, unless otherwise stated in figure legends. A Pearson correlation test was performed to explore associations between cell death and apoptotic signals (caspase 3/7 activation, ROS production, cytochrome c release). Differences were considered significant at P<0.05.

## Results

### Low doses of cereulide cause apoptosis in beta-cells, but not in other mammalian cell lines

**Figure 1:**
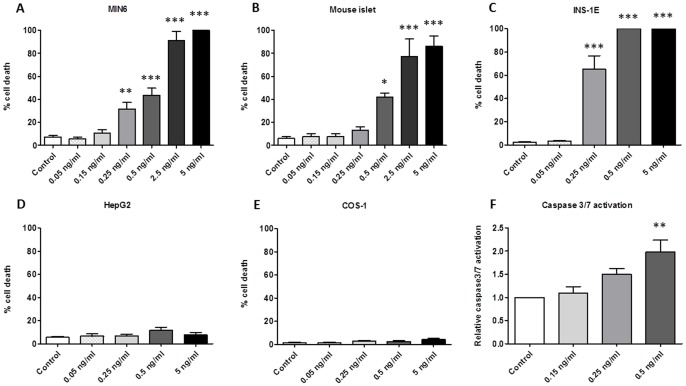
Cereulide causes apoptotic cell death in 3 beta-cell models, but not in other mammalian cell lines after 24 h exposure. Cell death rate was determined with Hoechst-PI staining for MIN6 cells (A, n = 6), mouse islets (B, n = 3), INS-1E (C, n = 3), COS-1 (D, n = 3) and HepG2 (E, n = 3), as well as caspase 3/7 activation (F, n = 3) in MIN6 cells after 24 h exposure to cereulide. Data are presented as mean ± SEM. * P<0.05, ** P<0.01 *** P<0.001 vs control.

31.6% of MIN6 cells were apoptotic when exposed to a concentration of 0.25 ng/ml for 24 h (P<0.01) and no cells survived exposure to 5 ng/ml (P<0.001 vs control, **figure 1A**). 72 h incubation with 0.15 ng/ml cereulide caused 47.5% apoptotic cell death versus 2.7% in non-exposed cells, while there were no viable MIN6 cells when exposed to 0.5 ng/ml for 72 h (both P<0.001, **table S2**). Whole mouse islets were similarly sensitive as the murine MIN6 cell line, with 0.5 ng/ml cereulide causing 42% cell death (vs 6% in control condition, P<0.05) and no surviving islets at 0.5 ng/ml exposure for 24 h (P<0.001, **figure 1B**). After 72 h of exposure, 0.15 ng/ml cereulide had no measurable effect, but 0.25 ng/ml caused apoptosis in 75% of the islets (P<0.05 vs control, **table S2**). The rat insulin producing INS-1E cell line appeared even more sensitive. Here, already 65.1% apoptotic cell death was observed at 0.25 ng/ml and 100% at 0.5 ng/ml after 24 h of exposure (both P<0.001 vs control, **figure 1C**). After 72 h, the cell death rate was 100% in INS-1E cells when exposed to 0.25 ng/ml (**table S2**). Importantly, no such effects were observed in two non-beta mammalian cell lines. There was no increased cell death in human liver HepG2 and monkey renal fibroblast COS-1 cells, after 24 h of exposure to the highest cereulide concentration (5 ng/ml) (**figure 1D and E**). In addition, even after 72 h of exposure to 5 ng/ml cereulide no increased cell death was observed in the HepG2 and COS-1 cell lines (**table S2**). A dose-dependent increase in caspase 3/7 activation was observed in MIN6 cells (1.97 fold in 0.5 ng/ml cereulide condition compared to control, P<0.01, **figure 1F**), which correlated with cell death (R = 0.653 and P<0.05).

### Cereulide impairs glucose stimulated insulin secretion

**Figure 2:**
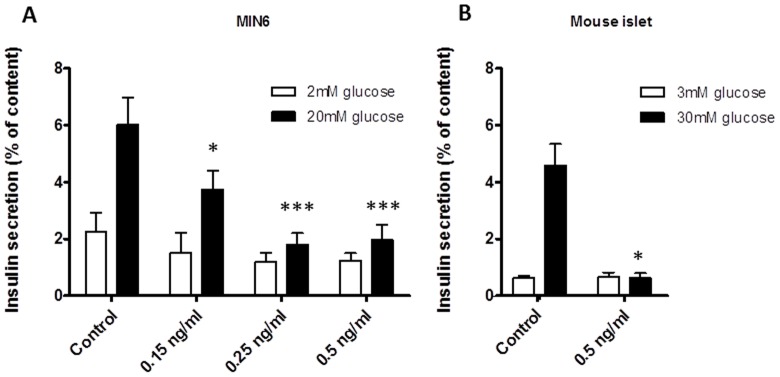
Cereulide impairs glucose-stimulated insulin secretion. MIN6 cells (A, n = 11) and whole mouse islets (B, n = 3) were exposed to cereulide for 24 h, and incubated with KREBS containing low (white bars) or high (black bars) concentrations of glucose. Insulin secretion is expressed as a percentage of total insulin content. Data are presented as mean ± SEM. * P<0.05, *** P<0.001 vs control (2 way ANOVA, followed by Bonferroni test for MIN6; student t test for islets).

Insulin secretion from control MIN6 cells was 2.2% of total insulin content at 2 mM glucose and increased to 6.0% when stimulated with 20 mM glucose. A dose dependent decrease in GSIS was observed following exposure to increasing cereulide concentrations. Exposure to 0.15 ng/ml cereulide significantly reduced the fraction of insulin secreted by MIN6 cells at 20 mM glucose (3.7% vs. 6.0% in the control stimulated cells) (P<0.05, **figure 2A**), and from exposure to 0.25 ng/ml cereulide on, the capacity to secrete insulin secretion upon glucose stimulation was almost completely lost (1.2% at 2 mM to 1.8% at 20 mM glucose). We confirmed this finding in whole mouse islets where GSIS capacity was also lost following 24 h exposure to 0.5 ng/ml cereulide (0.6% vs. 4.6% in the control islets at 30 mM) (P<0.05, **figure 2B**).

Basal insulin secretion did not differ between experimental conditions, nor did the total insulin content at the end of the experiment for the 0.15 ng/ml and 0.25 ng/ml cereulide condition in MIN6 cells, which is probably partly due to detection of insulin in unviable cells in the latter condition. Total insulin content decreased after exposure to 0.5 ng/ml cereulide in MIN6 cells and mouse islets, compatible with a reduced number of cells (P<0.05, **figure S1**).

### Cereulide induces mitochondrial and general proapoptotic stress markers

**Figure 3:**
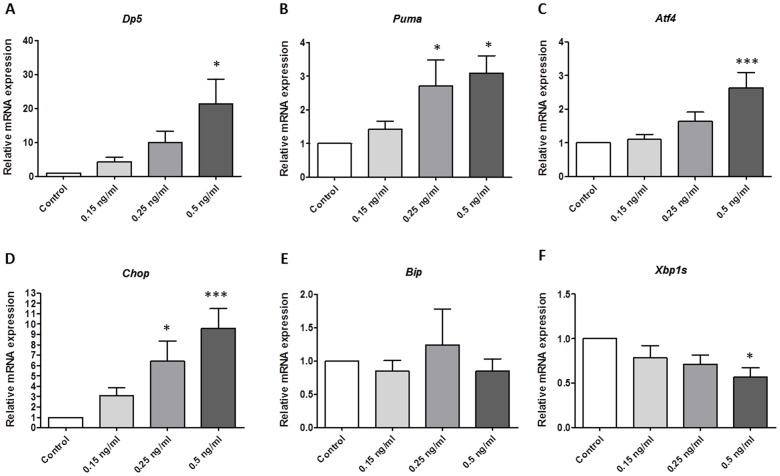
mRNA levels of intrinsic mitochondrial apoptosis and ER stress mediators are upregulated in MIN6 cells after 24 h of exposure to cereulide. Death protein 5 (*Dp5*, n = 4, A) and p53 upregulated modulator of apoptosis (*Puma*, n = 8, B) are dose dependently upregulated, as are activating transcription factor 4 (*Atf4*, n = 8, C) and CCAAT/-enhancer-binding protein homologous protein (*Chop*, n = 8, D), while binding immunoglobulin protein (*Bip*, n = 8, E) and spliced X-box binding protein are not (*Xbp1s*, n = 8, F). Data are presented as mean ± SEM (n 4–8). * P<0.05 vs control, *** P<0.001 vs control.

We observed a dose-dependent upregulation of mRNA levels of death protein 5 (*Dp5*; 21.4 fold of control at 0.5 ng/ml, P<0.05) and p53 upregulated modulator of apoptosis (*Puma*; 2.8 fold of control in 0.25 ng/ml condition; P<0.05, **figure 3A-B**), both members of the B-cell lymphoma (Bcl) 2 family, and known modulators of the intrinsic or mitochondrial pathway of cell death. There was no increase of *Bim, Bcl-XL* or *Mcl1* mRNA (**table S3**). However, mRNA levels of activating transcription factor 4 (*Atf4*) increased 2.6 fold in MIN6 cells after exposure to 0.5 ng/ml cereulide (P<0.01) and CCAAT/-enhancer-binding protein homologous protein (*Chop*) increased 7.3 fold even after exposure to the lower concentration of 0.25 ng/ml cereulide (both P<0.05). mRNA levels of Binding immunoglobulin protein (*Bip*) and of spliced X-box binding protein 1 (*Xbp1s*) did not increase following cereulide exposure (**figure 3C-F**). Moreover, additional Western blot experiments reveal that there is no phosphorylation of PERK, indicating that the rise in *Atf4* and subsequent *Chop* is not initiated directly by an endoplasmic signal (**figure S2**). In control cell lines, we observed a modest increase in mRNA levels of *Chop* after exposure to 0.5 ng/ml compared to control conditions: a 4.1 fold increase in HepG2 and 1.7 fold in COS-1 cells (both P<0.05). There was no significant increase in mRNA levels of *Atf4, Dp5, Puma* or *Xbp1s* (**figure S3**).

**Figure 4:**
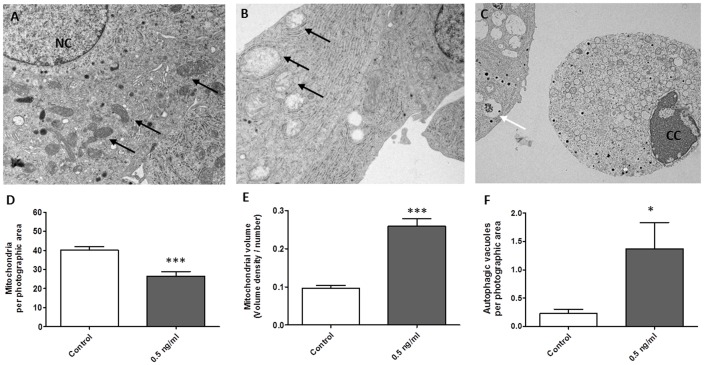
Cereulide disrupts normal mitochondrial structure in MIN6 cells. Electron microscopic evaluation of MIN6 cells in control condition (A) or after 24 h exposure to 0.5 ng/ml cereulide (B) (magnification 2500x). Arrows indicate normal mitochondria in control condition (A) or enlarged mitochondria with less cristae after cereulide exposure (B). Morphometric analysis confirmed fewer mitochondria (D) and swelling of the remaining mitochondria (E) in the exposed cells, as is calculated by dividing the mitochondrial area by the number of mitochondria. In the cereulide exposed condition, we noted cells with chromatin condensation (indicated by CC in panel 4C), compared to normal nuclei (indicated by NC in panel A). After cereulide exposure there were more autophagic vacuoles (F, indicated by white arrow in panel C) Data are presented as mean ± SEM. * P<0.05 vs control; *** P<0.001 vs control (student t test).

We analysed electron microscopy images from two independent experiments, after exposure to 0.5 ng/ml cereulide for 24 h or vehicle only. This confirmed the presence of markedly swollen mitochondria, with disrupted inner membrane structure, exclusively upon cereulide exposure (**figure 4A-B**). In the cereulide exposed cells we found apoptotic cells, (figure 4C) and more autophagic vacuoles (1.37 vs 0.23 in control condition, P<0.05) (figure 4F). More detailed morphometric analysis demonstrated less mitochondria in MIN6 cells exposed to 0.5 ng/ml cereulide as compared to control cells (26.5 vs 40.1 per photographic area, P<0.001), and confirmed the larger mitochondrial volume (0.26 vs 0.10 ml/100 ml, P<0.001) (**figure 4D-E**). No significant changes in ER volume or density, were observed following cereulide exposure.

### Cereulide negatively affects mitochondrial functioning

**Figure 5:**
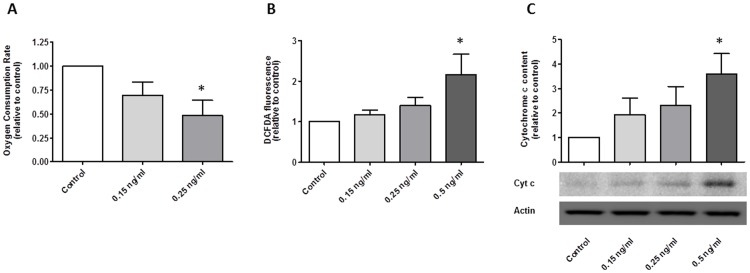
Cereulide negatively affects mitochondrial function in MIN6 cells after 24 h exposure. Oxygen consumption rate in basal conditions was reduced (A, n = 7). Cereulide caused a rise in reactive oxygen species, as shown by the increase in dichlorofluorescindiacetate fluorescence (B, n = 8). Cereulide exposure caused cytochrome c release into the cytoplasm (C, n = 4). Data are presented as mean ± SEM. * P<0.05 vs control, **P<0.01 vs control.

Analysis of basal oxygen consumption rate showed a marked and dose dependent decrease in respiration after exposure to cereulide. MIN6 cells exposed to 0.25 ng/ml for 24 h, consumed only 48% of the oxygen the control cells used (P<0.05, **figure 5A**). Addition of oligomycin or subsequent antimycin to the assay medium in the 0.25 ng/ml cereulide condition did not cause a further reduction in oxygen consumption rate, indicating that these cells have lost the capacity to generate ATP through the electron transport chain, even in resting conditions. We noted a dose dependent increase in ROS following exposure to cereulide (2.2 fold at 0.5 ng/ml cereulide exposure vs controls, P<0.05, **figure 5B**), again suggesting mitochondrial dysfunction. Finally, a gradual appearance of cytochrome c in the cytoplasm was seen after exposure to cereulide (3.71 fold at 0.5 ng/ml (P<0.05 vs control, **figure 5C**).

## Discussion

Our results demonstrate that even at very low concentrations, cereulide causes beta-cell dysfunction and ultimately beta-cell death, whereas non-beta-cell lines remain unaffected. Food inspections suggest that low concentrations of cereulide (4 ng/g food) are often present in take-away meals [17]. Although all our experiments were conducted in vitro, and the exact disposition of cereulide in vivo is not known, case reports of hepatic failure, splenic and serological presence of cereulide, support the hypothesis that cereulide can enter the portal and systemic circulation. While low exposure most probably does not lead to the typical systemic symptoms of food poisoning, our data demonstrate that even extremely low concentrations of cereulide are deleterious for the beta-cell's insulin secretory capacity. We observed a complete loss of insulin secretion following exposure to 0.5 ng/ml cereulide to mouse islets and merely 0.25 ng/ml in MIN6 cells. As there was no difference in basal insulin secretion nor in total insulin content in MIN6 cells after exposure to 0.15 ng/ml or 0.25 ng/ml cereulide, we can conclude that cereulide affects the insulin secretory machinery of the beta-cell directly, rather than affecting insulin synthesis. Given the mitochondrial toxicity seen in our experiments, with a decrease in basal oxygen consumption and increase in ROS and *Puma*, and given that beta-cells lack the potential for anaerobic glycolysis, the hindered mitochondrial production of ATP seems the most plausible explanation for the hampered insulin secretion capacity [25]. At higher concentrations of cereulide total insulin content did decrease, which confirms previous findings in porcine islets (1 ng/ml after 2 days) [7].

Beside the mitochondrial toxicity, cereulide also induced clear up-regulation of *Atf4* and *Chop*. Our initial suspicion of concomitant ER stress induction, could not be supported as there was no phosphorylation of the upstream activator PERK, no upregulation of Bip or Xbp1s mRNA and no dilation of the ER upon ultrastructural electron microscopy evaluation. Most likely, it is the oxidative stress caused by the dysfunctional mitochondria that activates the integrated stress response in this setting [26]. Importantly, the activation of the *Atf4-Chop* pathway causes an additional apoptotic signal leading to beta-cell loss [27]. Of note is that the antiapoptotic *Bcl-XL* and *Mcl1* mRNA levels were not increased, indicating that the pro-apoptotic signal is so prominent that the protective counter-regulatory responses are undermined. We also observed that slightly higher doses of cereulide effectively kill beta-cells, whereas the highly metabolic active human hepatocellular HepG2 and the structurally stronger monkey renal fibroblast COS-1 cell lines proved to be more resistant with no observed cell death, even after 72 h exposure to 5 ng/ml cereulide. In these cell lines there was a modest and isolated increase in mRNA levels of *Chop*, indicating that also the cells were stressed by cereulide, but apparently not to the irreversible point of apoptosis in the tested conditions.

In contrast to previous reports [28], our experiments show apoptotic cell death rather than necrotic cell death. Fluorescence microscopic evaluation with Hoechst-PI double staining showed pyknotic nuclei and nuclear morphology compatible with early to late apoptosis, and we noted abundant cells with chromatin condensation as well as autophagic vacuolization upon transmission electron microscopy. Activation of apoptotic pathways is further supported by the dose dependent cytochrome c release, capsase 3/7 activation, and *Chop* up-regulation. Of interest is that the toxic effects in the low concentration range in our experiments are similar to the data reported by the group of Hoornstra [28], and they also observed that other mammalian cells were unaffected at these low exposure levels. We hypothesize that cereulide's beta-cell specific toxicity is the result of the dependency of the beta cell on aerobic glycolysis through its mitochondria. Monocarboxylate transporters 1 (Mct1) have an extremely low expression in beta-cells to prevent the release of insulin in response to circulating pyruvate. As a result beta-cells might prove particularly sensitive to mitochondrial damage as they depend solely on aerobic glycolysis for their energy supply [11]. Our study has its limitations, as all experiments were conducted *in vitro*. However, this allowed us to explore the mechanisms involved in great detail, and we confirmed that toxicity is very similar in MIN6 cells and intact pancreatic islets. In addition, we confirmed cereulide's toxicity towards beta-cells in rats (INS-1E cell line) and whole mouse islets of Langerhans, suggesting the effect is not species specific.

To our knowledge, only one *in vivo* study in rodents and one in monkeys has been performed with cereulide, but they both focused on acute toxicity, emetic potential and mortality [29], [30]. Our data warrant to explore the *in vivo* beta-cell specific toxicity of cereulide in further studies.

We believe that the relevance of cereulide to human beta-cell disease, i.e. diabetes mellitus, is plausible, as it could be a link between the observed association between rice consumption and diabetes [17], [31], [32]. Furthermore, the etiology of puzzling clusters of fulminant non-autoimmune type 1 diabetes in Asia, remains to be elucidated but might entail exposure to food toxins such as cereulide [33], [34].

## Conclusion

Cereulide, a toxin frequently found in take-away meals, impairs glucose-stimulated insulin secretion and causes apoptosis in beta-cells of rats and mice at very low doses. The main underlying mechanisms of cereulide's toxicity involve mitochondrial damage with mitochondrial swelling, decreased oxygen consumption and increased ROS generation. Further study of the relevance of repeated exposure to low doses of cereulide is warranted, as it might play a role in the rise of the prevalence of diabetes worldwide.

## Supporting Information

Figure S1 **Total insulin content as measured in Glucose Stimulated Insulin Secretion (GSIS) experiments in MIN6 cells and mouse islets after 24 h exposure to cereulide.** Data are presented as mean ± SEM (for MIN6 n = 11, for islets n = 3). * P<0.05, (1 way ANOVA, followed by Dunnet test for MIN6; student t test for islets).
(TIF)

Figure S2 **Western blot for phosphorylated (P-PERK) and total protein kinase RNA-like endoplasmic reticulum kinase (PERK).** In MIN6 cells, PERK phosphorylation was not increased after 24 h exposure to cereulide 0.25–0.5 ng/ml cereulide, when compared to exposure with vehicle only (co). 1 µM thapsigargin (Tg), used as a positive control for ER stress, did increase phosphorylation of PERK. Representative sample from 3 independent experiments.
(TIF)

Figure S3 **mRNA levels of stress markers in HepG2 and COS-1 cells after 24 h exposure to cereulide.** Activating transcription factor 4 (*Atf4*), CCAAT/-enhancer-binding protein homologous protein (*Chop*), p53 upregulated modulator of apoptosis (*Puma*), Death protein 5 (*Dp5*), spliced X-box binding protein (*Xbp1s*). Data are presented as mean ± SEM (for HepG2 n = 5, for COS-1 n = 4). * P<0.05 vs control (student t test).
(TIF)

Table S1 **PCR primers used for qRT-PCR experiments.** FW denotes forward primer (5′–3′), RV denotes reverse primer (3′–5′); *Atf4* Activating transcription factor 4, *Bcl-XL* B-cell lymphoma extra-large, *Bim* Bcl2-interacting mediator of cell death, *Bip* Binding immunoglobulin protein, *Chop* CCAAT/-enhancer-bindingprotein homologous protein, *Dp5* Death protein 5, *Hprt* Hypoxanthine-guanine phosphoribosyltransferase, *Mcl1* myeloid cell leukemia sequence, *Puma* p53 upregulated modulator of apoptosis, *Rpl27* ribosomal protein L27, *Xbp1s* spliced variant of X-box binding protein 1.
(XLSX)

Table S2 **Percentage of dead cells in different cell lines and mouse islets after 72 h exposure to cereulide.** Data are presented as mean ± SEM (for MIN6 n = 5, for INS-1E n = 4, for HepG2, COS-1 and islets n = 3), * P<0,05 (1-way ANOVA, Dunnet post-test).
(XLSX)

Table S3 **mRNA levels of additional mitochondrial stress related genes in MIN6 cells after 24 h exposure to cereulide.** B-cell lymphoma-extra-large (*Bcl-XL*, n = 8), Bcl2-interacting mediator of cell death (*BIM*, n = 7), Myeloid cell leukemia sequence 1 (*Mcl1*, n = 8). Data are presented as mean ± SEM.
(XLSX)
