# Supplementary figures and images for: Foodborne Cereulide Causes Beta-Cell Dysfunction and Apoptosis
Source: PLoS One. 2014 Aug 13;9(8):e104866. doi: 10.1371/journal.pone.0104866 (PMC4132018; doi:10.1371/journal.pone.0104866)

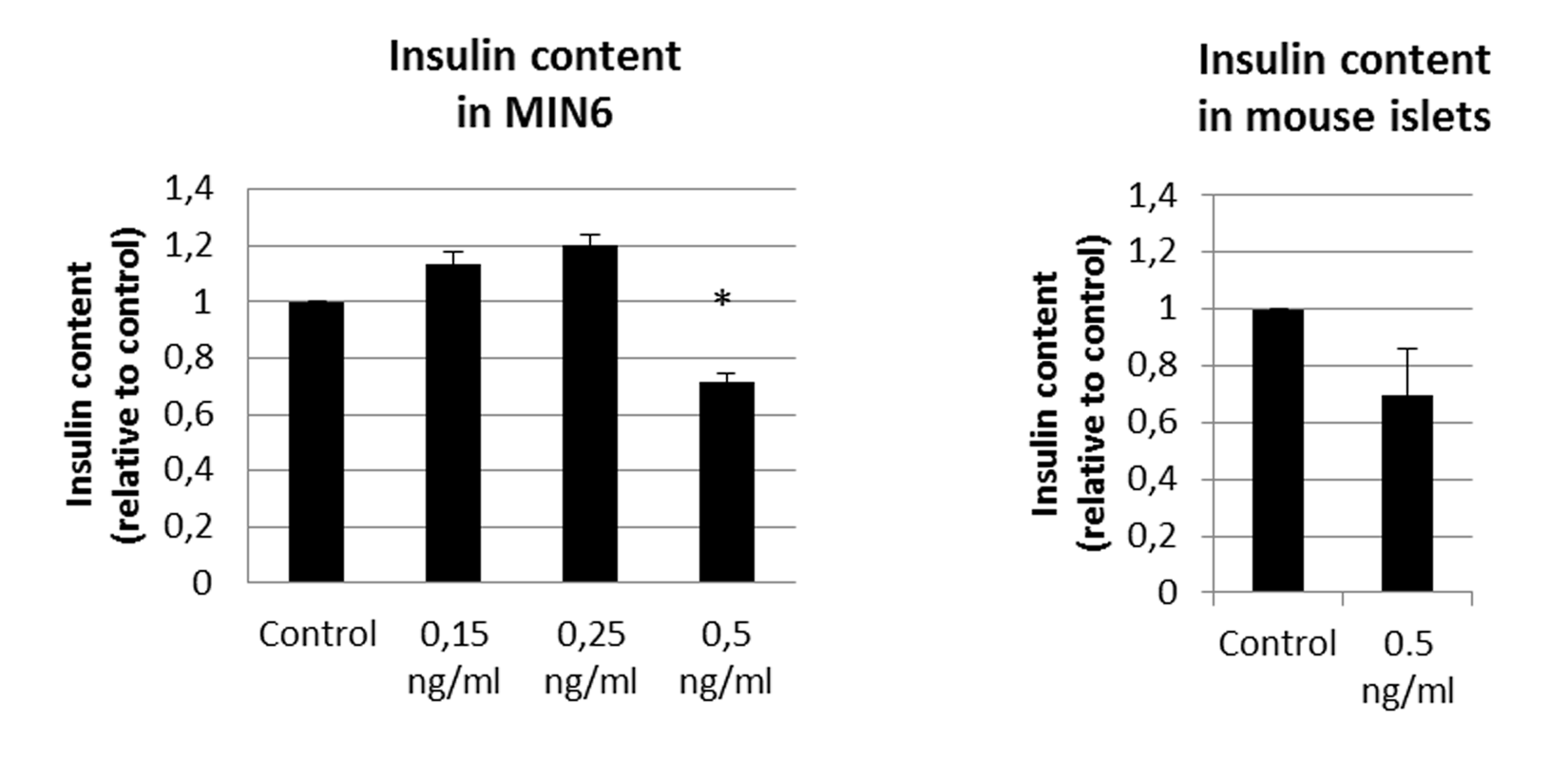

Supplement: Figure S1 — Total insulin content as measured in Glucose Stimulated Insulin Secretion (GSIS) experiments in MIN6 cells and mouse islets after 24 h exposure to cereulide. Data are presented as mean ± SEM (for MIN6 n = 11, for islets n = 3). * P<0.05, (1 way ANOVA, followed by Dunnet test for MIN6; student t test for islets). (TIF) [file pone.0104866.s001.tif]

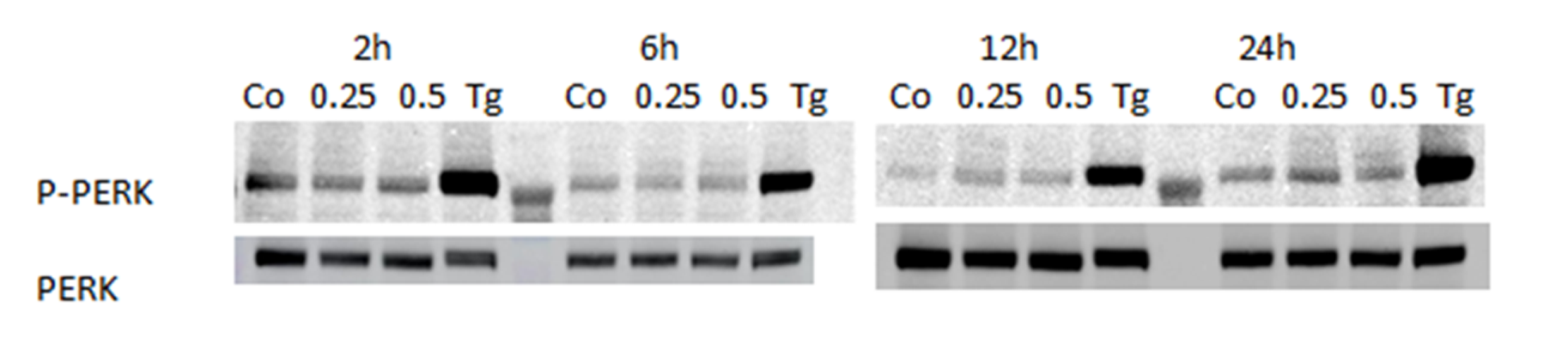

Supplement: Figure S2 — Western blot for phosphorylated (P-PERK) and total protein kinase RNA-like endoplasmic reticulum kinase (PERK). In MIN6 cells, PERK phosphorylation was not increased after 24 h exposure to cereulide 0.25–0.5 ng/ml cereulide, when compared to exposure with vehicle only (co). 1 µM thapsigargin (Tg), used as a positive control for ER stress, did increase phosphorylation of PERK. Representative sample from 3 independent experiments. (TIF) [file pone.0104866.s002.tif]

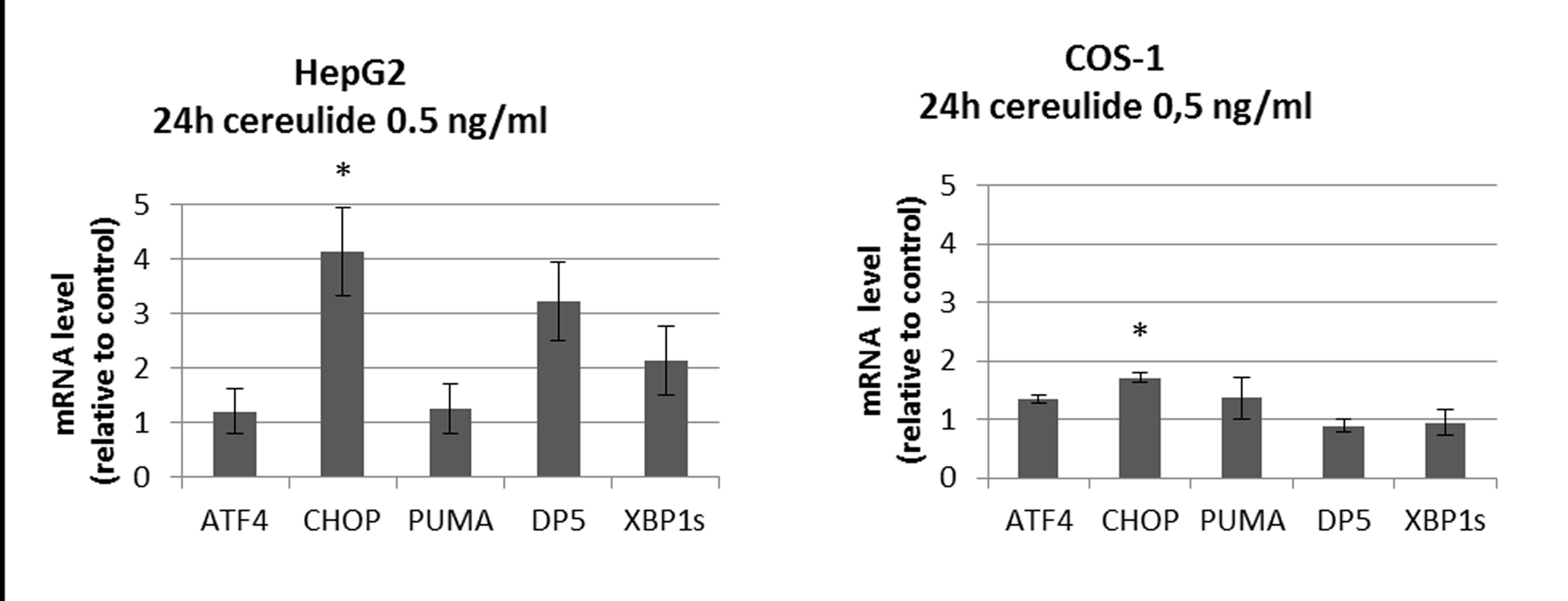

Supplement: Figure S3 — mRNA levels of stress markers in HepG2 and COS-1 cells after 24 h exposure to cereulide. Activating transcription factor 4 (Atf4), CCAAT/-enhancer-binding protein homologous protein (Chop), p53 upregulated modulator of apoptosis (Puma), Death protein 5 (Dp5), spliced X-box binding protein (Xbp1s). Data are presented as mean ± SEM (for HepG2 n = 5, for COS-1 n = 4). * P<0.05 vs control (student t test). (TIF) [file pone.0104866.s003.tif]
